# Supplementary material for: Experimental Evolution Under Biased Sex Ratios: Phenotypic and Genomic Responses in the Bulb Mite, Rhizoglyphus robini
Source: Genome Biol Evol. 2026 Jun 30;18(7):evag151. doi: 10.1093/gbe/evag151 (PMC13329799; doi:10.1093/gbe/evag151)
Supplement: evag151_Supplementary_Data [file evag151_supplementary_data.zip › Supplementary information.pdf]

Supplementary information

Experimental evolution under biased sex ratios: phenotypic and genomic responses in the bulb mite, Rhizoglyphus robini

Chmielewski S., Parrett J.P., Konczal M., Radwan J.

List of supplementary figures

Figure S1. Estimates of effective population sizes

Figure S2. Coverage distribution

Figure S3. Manhattan plots of SNPs which significantly diverged in both treatments between ancestral and evolved populations

Figure S4. Distribution of haplotype length

Figure S5. Allele counts from the only SNP that was diverged between treatments in the evolved lines in GLM.

Figure S6. Replicate-specific genomic responses to experimental evolution.

List of supplementary tables (attached separately as an Excel file)

Table S1. Mean values and standard errors for autosomal genetic diversity measures

Table S2. Summary of sequencing yield per sample

Table S3. Line-specific estimates of effective population sizes

Table S4. Functional description and allele frequency change in SNPs selected in opposite direction between male- and female-biased lines which were significant in both treatments.

Table S5. Haplotype-averaged allele frequency changes.

Table S6. SNP counts per chromosome for each line and generation.

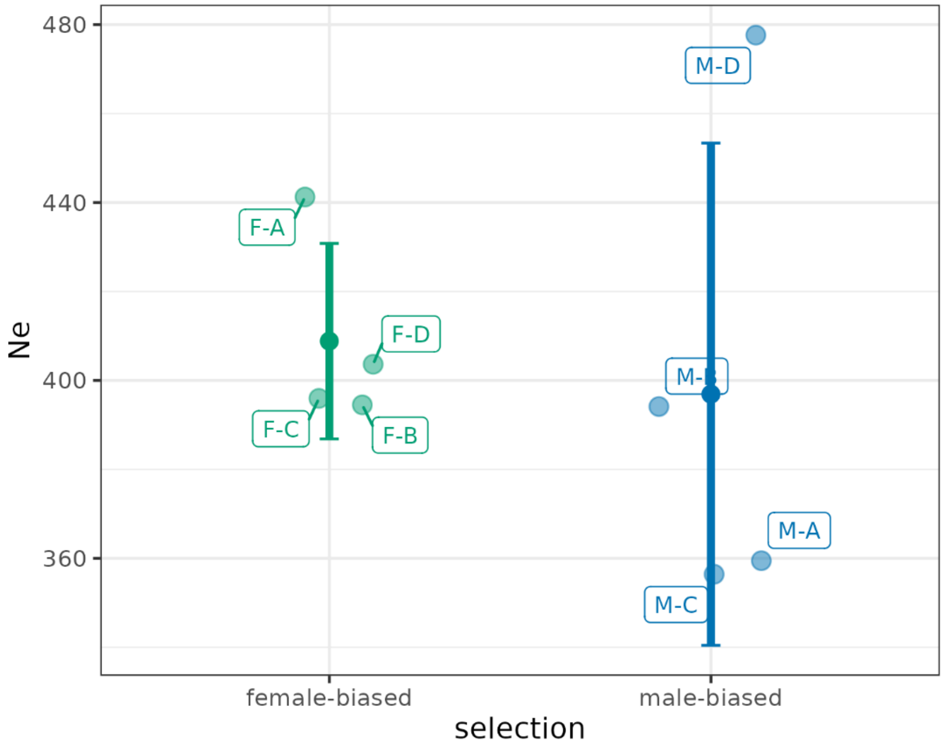

Figure S1. Estimates of effective population sizes based on allele frequency change between ancestral and evolved lines.

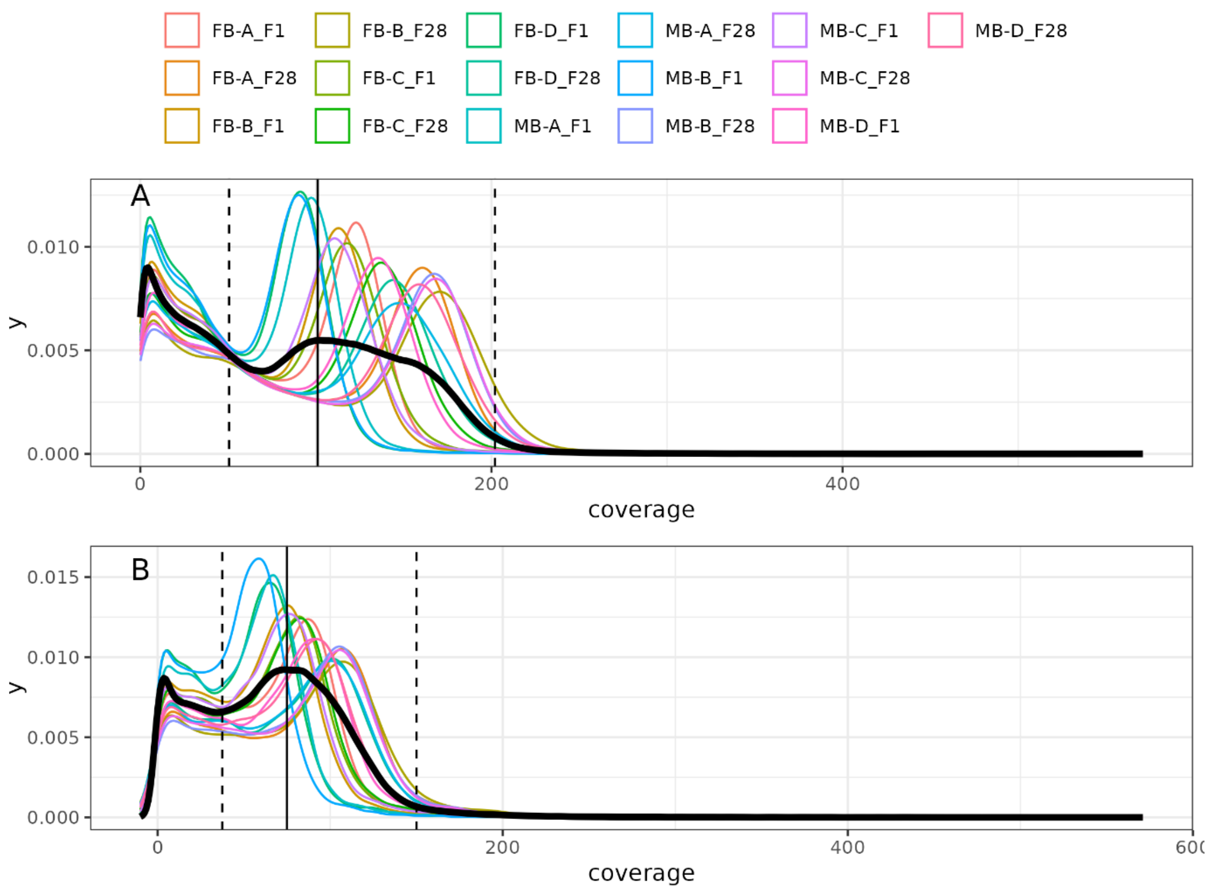

Figure S2. Distribution of coverage for autosomal (A) and sex chromosome (B) loci. The black smoothed line indicates the average coverage across lines. The vertical solid line represents the target coverage, while the dashed lines indicate the range of informative coverage, spanning from 50% to 200% of the target coverage. In panel A, the long tail of the coverage distribution has been trimmed to the maximum coverage value of the sex chromosome (571X), removing 12 positions with high coverage values.

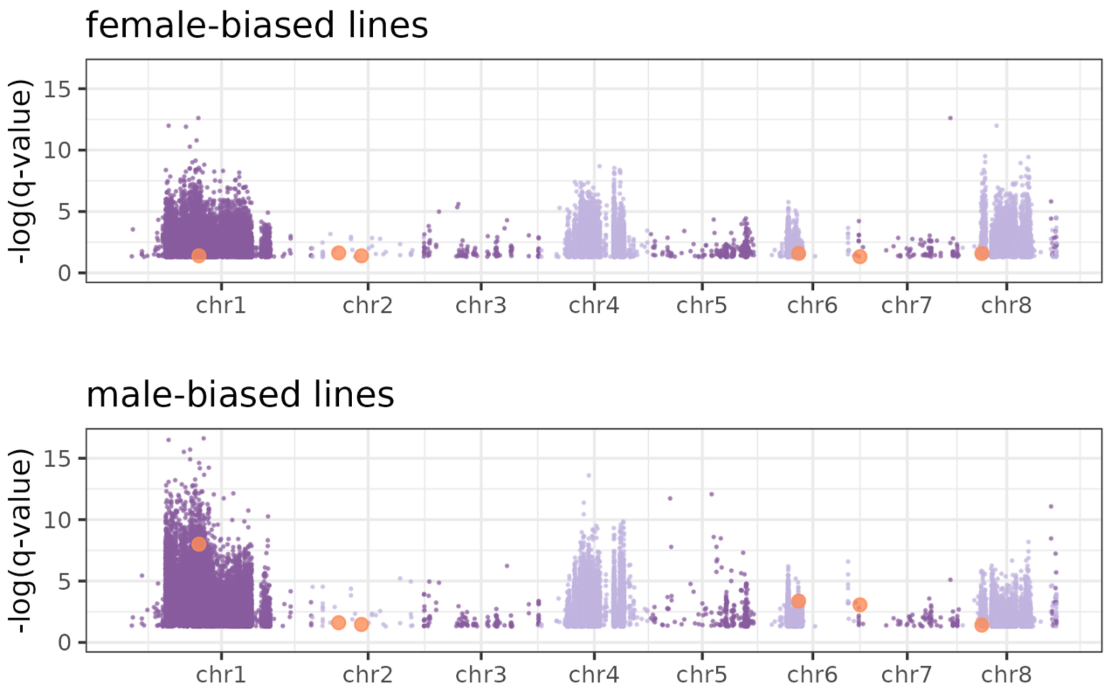

Figure S3. Manhattan plots of SNPs which significantly diverged in both treatments between ancestral and evolved populations. Plots show the *q* value of ACER test performed on either female-biased lines (upper panel), or male-biased lines (lower panel). All SNPs, besides these highlighted in orange, changed their frequency concordantly in both treatments. SNPs highlighted in orange changed their frequency in opposite direction between female- and male-biased lines. Colours refer to the Figure 4.

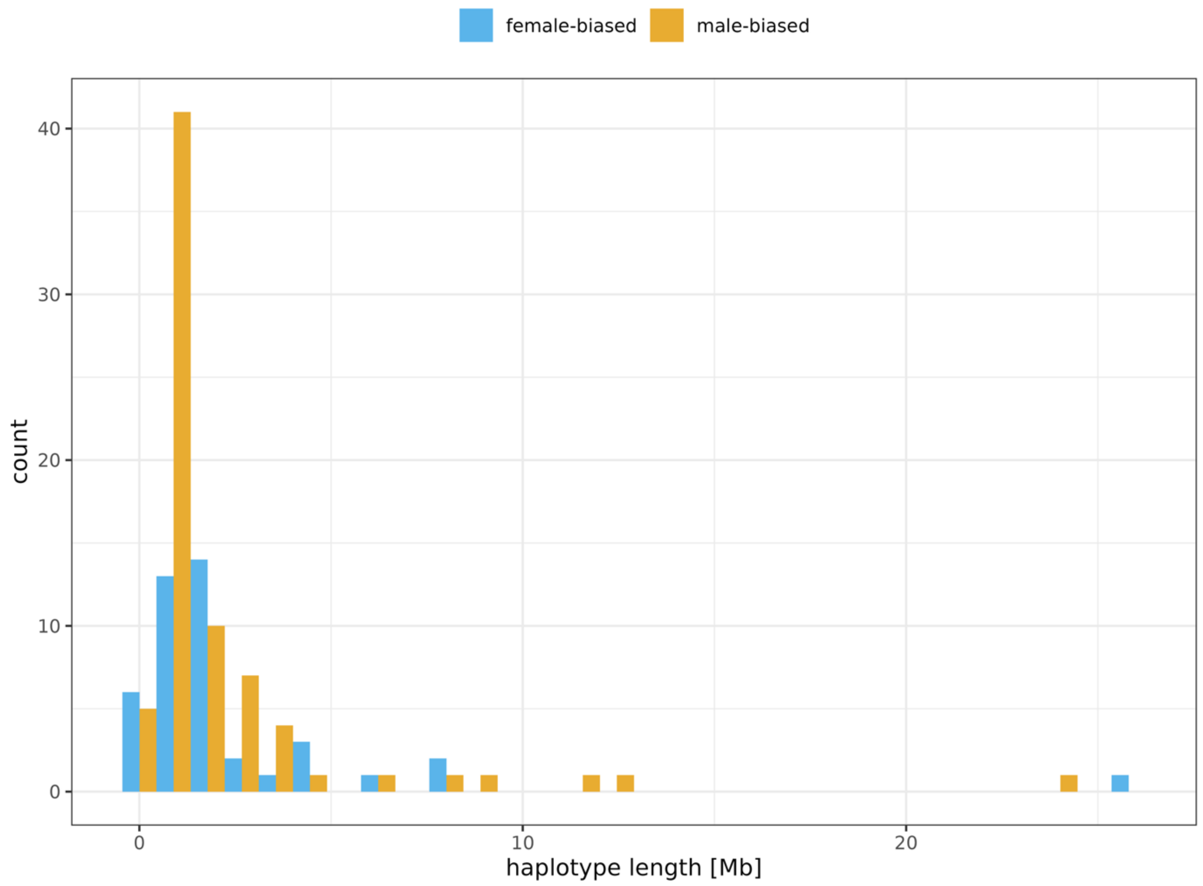

Figure S4. Distribution of haplotype length.

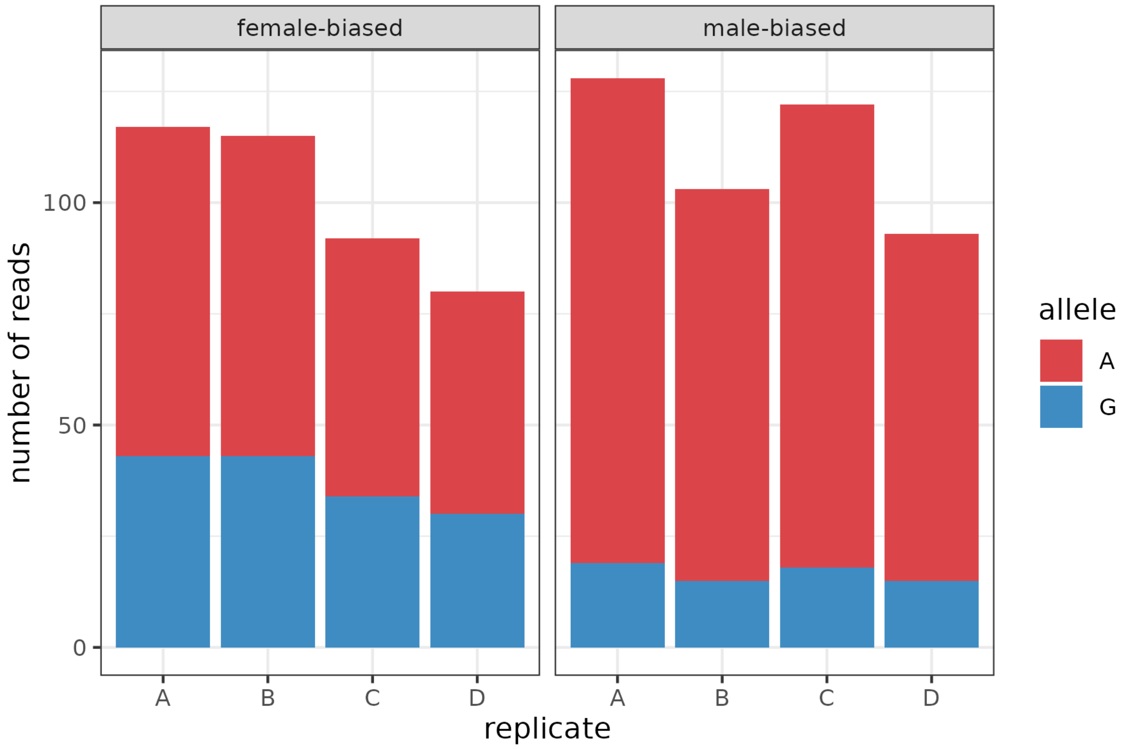

Figure S5. Allele counts from the only SNP that was diverged between treatments in the evolved lines, as indicated by the GLM.

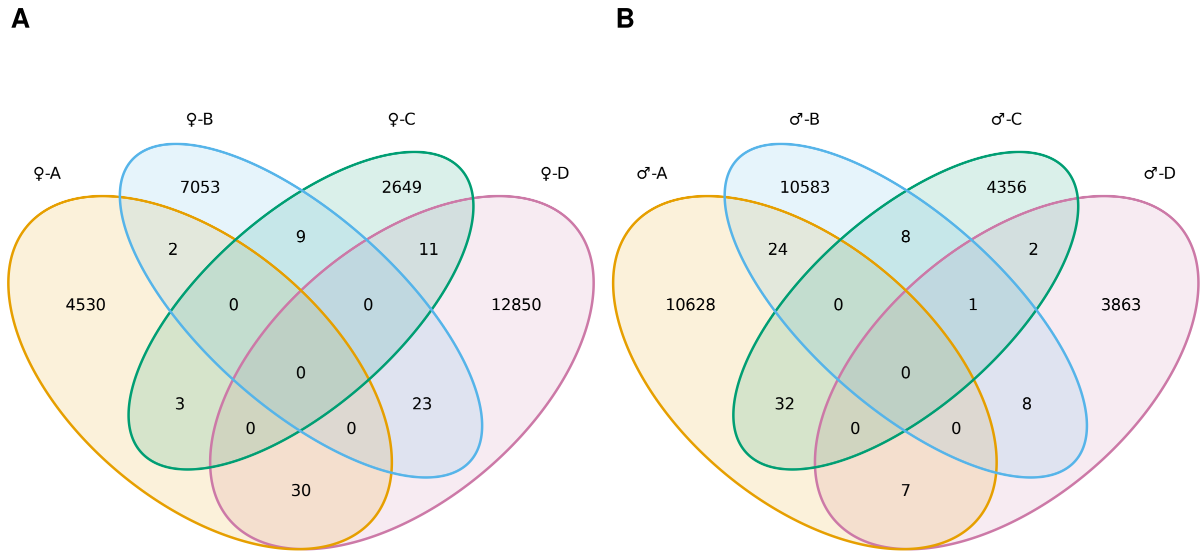

Figure S6. Replicate-specific genomic responses to experimental evolution. Venn diagrams show overlap among replicate lines in SNPs that changed significantly in frequency between ancestral and evolved populations when each line was analysed separately with the adapted chi-square test. A: female-biased lines (FB-A to FB-D), and B: male-biased lines (MB-A to MB-D). Numbers indicate counts of significant SNPs unique to a given line or shared among replicate lines within a treatment.
